# Supplementary material for: A mixed methods study of community-based health insurance enrollment trends and underlying challenges in two districts of northeast Ethiopia: A proxy for its sustainability
Source: PLoS One. 2022 Aug 29;17(8):e0266583. doi: 10.1371/journal.pone.0266583 (PMC9423627; doi:10.1371/journal.pone.0266583)
Supplement: Attachment — Submitted filename: Response to Reviewers.docx [file pone.0266583.s002.docx]

**Responses to Reviewers**

Dear editor, we are enclosing herewith the second revised version on the manuscript entitled: “A mixed methods study of community-based health insurance enrollment trends and underlying challenges in two districts of northeast Ethiopia: a proxy for its sustainability” with reference “PONE-D-22-08512R1”.

We are grateful to the handling editor Dr Pablo Villalobos Dintrans for his comments and effective facilitation of the review process. Our responses to the concerns raised during the second review process are presented below.

**Editor Comments:**

1. I think the authors did a good job addressing the comments. I still would like a more sound discussion on the methods and the way this study can be viewed as "mixed methods". These references can be helpful for better defined what is a mixed method study, what type was used, and why it makes sense for this particular study and its aim:

--- Thank you very much for the comment. We thank also for the materials you shared with us. We tried to made some revisions on the methods section as to the comments [Line 176-187] - Manuscript with Track Changes

2. Also, I think you need a better response to comment 16 (results) [16. Table 1, Figure 1. Is there really a trend or pattern in the data displayed? Results seem different for the two districts, is it possible from the qualitative analysis to further explain why?]. This is exactly what I would expect to see the advantage of using a mixed method study, in which you explain some of the observed (quant) results using your methodology (qual). Please expand this discussion further.

--- Thank you again for raising this issue. In addition to what we have do during the first revision, we try to expand the discussion further [Line 642-646] - Manuscript with Track Changes
